# *Cannabis sativa* extracts protect LDL from Cu^2+^-mediated oxidation

**DOI:** 10.1186/s42238-020-00042-0

**Published:** 2020-10-15

**Authors:** Bruno Musetti, Helena González-Ramos, Mercedes González, Edward M. Bahnson, Javier Varela, Leonor Thomson

**Affiliations:** 1 Laboratorio de Enzimología, Instituto de Química Biológica, Facultad de Ciencias, Universidad de la República, Iguá 4225, 11400 Montevideo, Uruguay; 2 Grupo de Química Orgánica Medicinal, Facultad de Ciencias, Universidad de la República, Iguá 4225, 11400 Montevideo, Uruguay; 3 Division of Vascular Surgery, Department of Surgery, and Department of Cell Biology & Physiology, University of North Carolina at Chapel Hill, Chapel Hill, NC 27599 USA; 4 Center for Nanotechnology in Drug Delivery, University of North Carolina at Chapel Hill, Chapel Hill, NC 27599 USA

**Keywords:** *Cannabis sativa*, Maturation, Phytocannabinoids, Hemp, Low-density lipoprotein, Oxidation, Atherosclerosis

## Abstract

**Background:**

Multiple therapeutic properties have been attributed to *Cannabis sativa.* However, further research is required to unveil the medicinal potential of Cannabis and the relationship between biological activity and chemical profile.

**Objectives:**

The primary objective of this study was to characterize the chemical profile and antioxidant properties of three varieties of *Cannabis sativa* available in Uruguay during progressive stages of maturation.

**Methods:**

Fresh samples of female inflorescences from three stable *Cannabis sativa* phenotypes, collected at different time points during the end of the flowering period were analyzed. Chemical characterization of chloroform extracts was performed by ^1^H-NMR. The antioxidant properties of the *Cannabis sativa* extracts, and pure cannabinoids, were measured in a Cu^2+^-induced LDL oxidation assay.

**Results:**

The main cannabinoids in the youngest inflorescences were tetrahydrocannabinolic acid (THC-A, 242 ± 62 mg/g) and tetrahydrocannabinol (THC, 7.3 ± 6.5 mg/g). Cannabinoid levels increased more than twice in two of the mature samples. A third sample showed a lower and constant concentration of THC-A and THC (177 ± 25 and 1 ± 1, respectively). The THC-A/THC rich cannabis extracts increased the latency phase of LDL oxidation by a factor of 1.2–3.5 per μg, and slowed down the propagation phase of lipoperoxidation (IC_50_ 1.7–4.6 μg/mL). Hemp, a cannabidiol (CBD, 198 mg/g) and cannabidiolic acid (CBD-A, 92 mg/g) rich variety, also prevented the formation of conjugated dienes during LDL oxidation. In fact, 1 μg of extract was able to stretch the latency phase 3.7 times and also to significantly reduce the steepness of the propagation phase (IC_50_ of 8 μg/mL). Synthetic THC lengthened the duration of the lag phase by a factor of 21 per μg, while for the propagation phase showed an IC_50_ ≤ 1 μg/mL. Conversely, THC-A was unable to improve any parameter. Meanwhile, the presence of 1 μg of pure CBD and CBD-A increased the initial latency phase 4.8 and 9.4 times, respectively, but did not have an effect on the propagation phase.

**Conclusion:**

Cannabis whole extracts acted on both phases of lipid oxidation in copper challenged LDL. Those effects were just partially related with the content of cannabinoids and partially recapitulated by isolated pure cannabinoids. Our results support the potentially beneficial effects of *Cannabis sativa* whole extracts on the initial phase of atherosclerosis.

## Introduction

Cannabis has been used for medicinal purposes for centuries (Pain 2015). Current changing legal frameworks concerning cannabis (Gould 2015) must come together with scientific data to support the scope of therapeutic benefits and risks, thus providing rigorous knowledge to the medical community and decision makers.

Inflammation is part of a protective response given by the immune system. Specifically, the innate immune system has been highly conserved through evolution to respond to harmful stimuli, such as pathogens or irritants. During inflammatory processes, macrophages and neutrophils produce oxygen and nitrogen derived oxidants (Iles and Forman 2002). Although these molecules are involved in relevant signaling and defense processes (Brüne et al. 2013), if inflammation persists, intracellular and extracellular molecules will become oxidized, leading to endothelial dysfunction and tissue damage (Mittal et al. 2014).

**Fig. 1:**
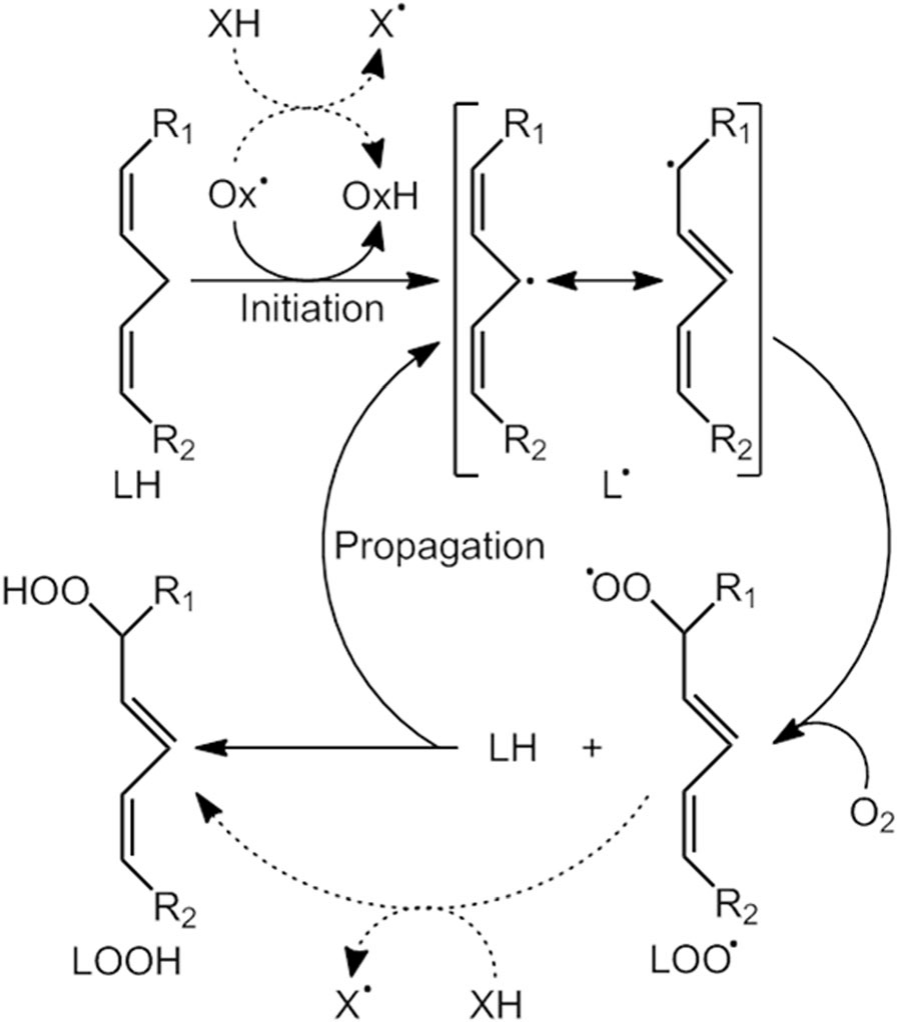
Mechanism of unsaturated fatty acid oxidation. In the first step, the unsaturated fatty acid (LH) is attacked by an oxidant (Ox^●^), abstracting a bis-allylic hydrogen and giving rise to an alkyl radical (L^●^), this radical can react with O_2_ generating a peroxyl radical (LOO^●^). The propagation phase is triggered and perpetuated by the reaction of L^●^ and LOO^●^ with reduced lipid molecules. Antioxidants (XH) can act preventing the initiation step or participating in the termination phase of lipid oxidation

In atherosclerosis, oxidized LDL (oxLDL) drives the formation of foam cells and acting as a damage signal stimulates the synthesis of cytokines and other chemotactic factors triggering an immune response in the subendothelial space (Libby et al. 2002; Moore and Tabas 2011). Oxidation of lipids in LDL proceeds as with other polyunsaturated fatty acid (PUFA) rich complexes through three main phases: initiation, propagation and termination (Fig. 1) (Yin et al. 2011). The initiation phase is promoted by a highly reactive molecule, frequently bearing a free radical, able to react with enough energy to exceed the dissociation energy of the allylic bond. This reaction causes the abstraction of a hydrogen and the formation of an alkyl radical (L^•^). The alkyl radical is stabilized by resonance by adjacent groups, forming conjugated double bonds (conjugated dienes) that exhibit a characteristic maximal absorption at 234 nm. Molecular oxygen rapidly reacts with alkyl radicals to form a lipoperoxyl radical (LOO^•^), an important intermediate in the propagation chain; once this radical is formed, the chain of oxidative reactions will continue by abstracting a hydrogen atom from other nearby alkyl groups (Yin et al. 2011). The initiation phase is characterized by a “latency phase” that is determined by the reactivity of the involved lipids, and in the case of the plasma lipoproteins, will vary with the presence, concentration and reactivity of endogenous antioxidants (Schuster et al. 1995). The propagation phase is characterized by cyclic reactions of peroxyl (LOO^•^) and alkyl (L^•^) radicals with newly recruited unsaturated fatty acids (LH). Several competing termination reactions exist, including the bimolecular reaction between two fatty acid derived radicals (Eq. 1–3) or between a propagation phase intermediate and an antioxidant molecule (Halliwell 1995).

1 $$ {\mathrm{L}}^{\bullet }+{\mathrm{L}}^{\bullet}\to \mathrm{LL} $$

2 $$ {\mathrm{LOO}}^{\bullet }+{\mathrm{LOO}}^{\bullet}\to \mathrm{LOOL}+{\mathrm{O}}_2 $$

3 $$ {\mathrm{L}}^{\bullet }+{\mathrm{L}\mathrm{OO}}^{\bullet}\to \mathrm{LOOL} $$

In fact, some antioxidant molecules (XH in Fig. 1), whether endogenous or not, have the capacity to intervene in one or both phases of lipid oxidation, preventing or delaying the appearance of lipid oxidation products.

The antioxidant and anti-atherogenic properties of *Cannabis sativa* extracts has been previously reported (Borges et al. 2013; Walsh et al. 2010). In fact, it has been shown that sub-psychotropic concentrations of THC inhibited the progression of atherosclerotic lesions in apo-E knock out mice, decreasing the production of cytokines and inhibiting cell proliferation and chemotaxis (Steffens et al. 2005). In addition, selective activation of the cannabinoid receptor 2 (CBR2) decreased the expression of CD36 scavenger receptor, and the production of TNF-α, IL-12 and IL-10, and reduced cellular oxLDL accumulation (Chiurchiù et al. 2014). Moreover, the administration of THC to STZ-diabetic rats was also effective in reducing blood glucose and attenuating serum markers of oxidative stress and lipid peroxidation (Vella et al. 2017).

While the main focus in cannabis research has been given to tetrahydrocannabinol (THC), its precursor, tetrahydrocannabinolic acid (THC-A) has been much less studied. The absence of psychoactive effects, positions THC-A as an interesting targets for medicinal development (Burstein 1999). This study was designed to explore the evolution of the molecular composition of the inflorescences of *Cannabis sativa* during maturation and the relationship between composition and biological activity. As a measure of biological activity, the capacity of the extracts to prevent LDL oxidation was explored and compared with isolated phytocannabinoids and hemp, a variety devoid of the psychotropic cannabinoids.

## Materials and methods

### Materials

Δ^9^-tetrahydrocannabinol (THC), Δ^9^-tetrahydrocannabinolic acid (THC-A), cannabidiol (CBD) and cannabidiolic acid (CBD-A), high quality reference standards for research (≥ 98% purity), were purchased from Echo Pharmaceuticals (Leiden, The Netherlands). The inflorescences of *Cannabis sativa* were provided by a registered cannabis club (#42) based in Montevideo, Uruguay. The sample of hemp was provided by International Cannabis Corporation (ICC), Uruguay. All other reagents were from Sigma-Aldrich (St. Louis, MO, USA) unless otherwise specified.

### Sampling and preparation of extracts

Fresh samples of female inflorescences were collected at different times during the end of the flowering period from three stable *Cannabis sativa* phenotypes, named Strawberry (SC), Exodus Cheese (EC) and Magma (Mag). The collection of fresh samples was performed in 3 different occasions, with a period of 5 days between the first (samples 1) and the second collection (samples 2), and 10 days between the second and third collection (samples 3) coincident with the regular harvest time. A fourth sample (samples 4) of dried floral parts was also collected, 30 days after harvest. The samples were observed using a digital microscope (Dino-Lite, CA). Extracts of duplicate samples (1 g each) were obtained by dynamic maceration for 20 min in chloroform (CHCl_3_). The resulting extracts were filtered and then brought to dryness using a rotary evaporator. Immediately before use the samples were solubilized in DMSO at the desired concentration. A dried sample of hemp inflorescences, processed under the same conditions, was included for comparative purposes.

### ^1^H-NMR quantification

Dried extracts (20 mg) were dissolved in 600 μL of deuterated chloroform (CDCl_3_) and the internal standard tetramethylsilane (TMS, 0.25 M) was added. Spectra were acquired using an NMR instrument (Bruker DPX 400 MHz), 64 scans were used, requiring 10 min and 26 s of acquisition with the following parameters: 0.16 Hz/point, pulse width (PW) = 30 ° (11.3 ms), and relaxation time (RD) = 1.5 s. 8; 39; 83 (Happyana and Kayser 2016).

### UV/Vis spectra

The absorption spectra of *Cannabis sativa* extracts were recorded using a Cary 60 UV-Vis spectrophotometer (Agilent Technologies, CA). Increasing concentration of the extracts (0.1–2 mg/mL) were diluted in DMSO: phosphate buffer (100 mM, pH 7.4) 20:1 (v:v) and the absorbance between 200 and 600 nm was recorded against a blank containing the same amount of DMSO. Extinction coefficients at the two absorbance peaks (298 and 257 nm) were obtained by linear regression.

### Isolation of LDL from human plasma

Human plasma was obtained from blood donors after informed consent at the Department of Transfusion Medicine, Hospital de Clínicas, Facultad de Medicina, Universidad de la República, Uruguay. The procedures were in accordance with the Helsinki’s Declaration (World Medical Association. 2001) and the research protocol approved by the Institutional Committee. Each blood donor was informed of their right to refuse, the relevance of the investigation and the privacy (identity protection) assured, while an informed consent form was signed by each donor. A total of three blood samples from healthy male volunteers (age 36 ± 11 years) were processed. Total blood (450 mL) was collected in primary bags containing 63 mL of anticoagulant solution CPD (129 mM dextrose, 105 mM citrate and 16 mM phosphate, Terumo Corporation, Tokyo, Japan), centrifuged using a Roto Silenta 63RS transfusion bag centrifuge (Hettich, Germany) at 2.200 rpm at 20^o^ C. Plasma was obtained in secondary bags and preserved at -20^o^ C until use, in less than a week. Human LDL was isolated using already published techniques (Chapman et al. 1981). Briefly, human plasma samples were mixed with KBr (0.28 g/mL) and placed in ultracentrifuge tubes up to ~ 40% of their volume, the remaining volume was filled with 0.15 M NaCl slowly added against the tube walls. The samples were centrifuged at 300,000 g for 90 min at 4 °C. The orange layer appearing in the superior third was collected and dialyzed at 4^o^ C against 100 mM phosphate buffer, pH 7.4. Protein concentration of the LDL fraction was determined at 280 nm using an extinction coefficient of 1 (mg/mL)^− 1^.cm^− 1^.

### Analysis of lipid oxidation

The most common method for the determination of antioxidant properties of natural compounds is the LDL oxidation assay (Kiokias et al. 2018). LDL is isolated from human plasma, and oxidation is induced by Cu^2+^ ions and is monitored spectrophotometrically via the change of absorption at 234 nm due to the formation of conjugated dienes (Esterbauer et al. 1989; Pinchuk and Lichtenberg 1996). Since the kinetics of LDL oxidation by cupper is well defined, the method allows to study changes in lag phase, due to endogenous antioxidants, and the exponentially growing propagation phase. Plasma LDL fractions (0.1 mg/mL) were equilibrated at 37 °C in 100 mM phosphate buffer, pH 7.4, in the absence and presence of increasing concentrations of *Cannabis sativa* extracts (0.5–5 μg/mL) and pure phytocannabinoids (0.025–1.25 μg/mL). Lipoprotein oxidation was triggered by CuSO_4_ (50 μM) and the formation of conjugated dienes was followed at 234 nm (Esterbauer et al. 1989; Pinchuk and Lichtenberg 1996), using a Cary 60 UV-Vis spectrophotometer (Agilent Technologies, CA), against a blank containing buffer and the same percentage of DMSO. Since the extracts were solubilized in DMSO, to account for potential interferences, exactly the same volume of DMSO (0.2% of the final volume) was present in every condition. The data obtained were adjusted to a sigmoid function (Eq. 4)

4 $$ y={A}_1+\left({A}_2-{A}_1\right)\ \left(1+{10}^{\left(\left(\mathit{\log}{IC}_{50}-x\right)S\right)}\right) $$

Where A_1_ and A_2_ represent the minimum and maximum absorbance, respectively; while IC_50_ and S represent the half-life and the rate of change (slope) of the propagation phase, respectively. The duration of the initiation phase (latency) was measured from the addition of the oxidant until the time where the absorbance riches 10% of the total span (A_2_-A_1_). The latency ratio, calculated as the relationship between the length of the initiation phase in the presence and the absence of extracts, was plotted against the extract added in μg; the slope of this linear plot was reported as the antioxidant capacity.

The ability to interrupt the reaction chains during the propagation phase, named here as Protection, was calculated from the ratio between the slope (S) obtained in the presence (S_e_) and in the absence (S_0_) of the extracts and presented as percentage (Eq. 5):

5 $$ Protection\ \left(\%\right)=100-\frac{S_e}{S_o}x\ 100 $$

An IC_50_ for each extract was determined from the sigmoidal fitting of a Protection vs. log dose-response curve.

### Statistical analysis

Data were analyzed using GraphPad Prism 6.0 (GraphPad Software, La Jolla, CA). Correlations were analyzed by linear regression. Statistical analyses were performed by two-way analysis of variance (ANOVA) and Sidak-Bonferroni’s post hoc to perform multiple comparison test. Differences with *p* < 0.05 were considered statistically significant.

## Results

### Extracts preparation and chemical characterization

**Fig. 2:**
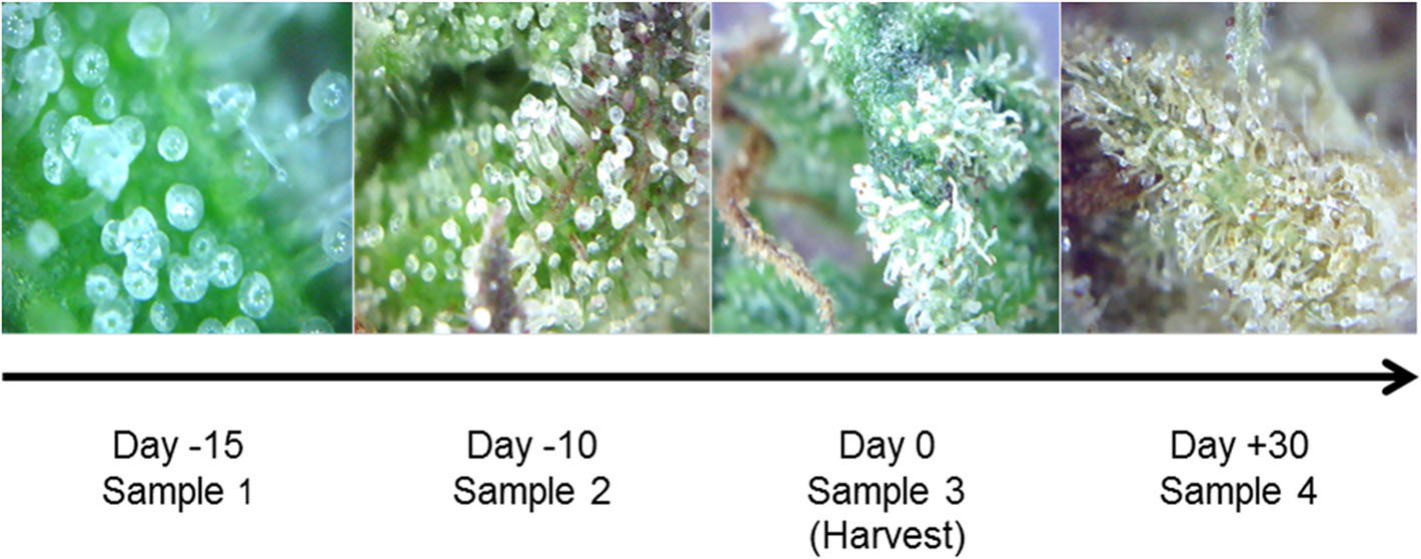
Temporal evolution of the glandular trichomes. *Cannabis sativa* inflorescences were captured with a digital microscope immediately before the collect for analysis at 15 (sample 1), 10 (sample 2) and 0 (sample 3) days before regular harvest time and trichomes dried for 30 days after harvest (sample 4)

The maturation of the inflorescence leads to an increase of the number and size of the trichomes (Fig. 2). Coincident with these morphological changes, the mass of material obtained per gram of tissue processed augmented significantly, denoting the accumulation of metabolites in the final period of maturation (Table 1), in concordance with previous reports (Happyana and Kayser 2016). In agreement to the popular knowledge concerning optimal harvest time, the increase in metabolites is indeed evidenced through changes in the coloration of glandular trichomes, which began mostly transparent, changed to a denser white color, and finally turned to an amber hue (Fig. 2). As result of water loss, a significant increase in the yield was found for the extractions carried out from most dried samples (Table 1).

**Table 1:** Extraction yields

		Yield (%)^**a**^		
Sample		SB	EC	Mag
**1**	day −15	11.2 ± 1.1	8.0 ± 1.2	10.4 ± 0.6
**2**	day − 10	13.3 ± 1.9	13.1 ± 1.3	15.5 ± 1.4
**3**	harvest	16.1 ± 2.4	14.8 ± 2.2	15.0 ± 2.0
**4**	dried	26.2 ± 1.4	26.4 ± 3.2	16.0 ± 0.8

^a^ Yields were expressed as the percentage of the total mass obtained from each plant

**Fig. 3:**
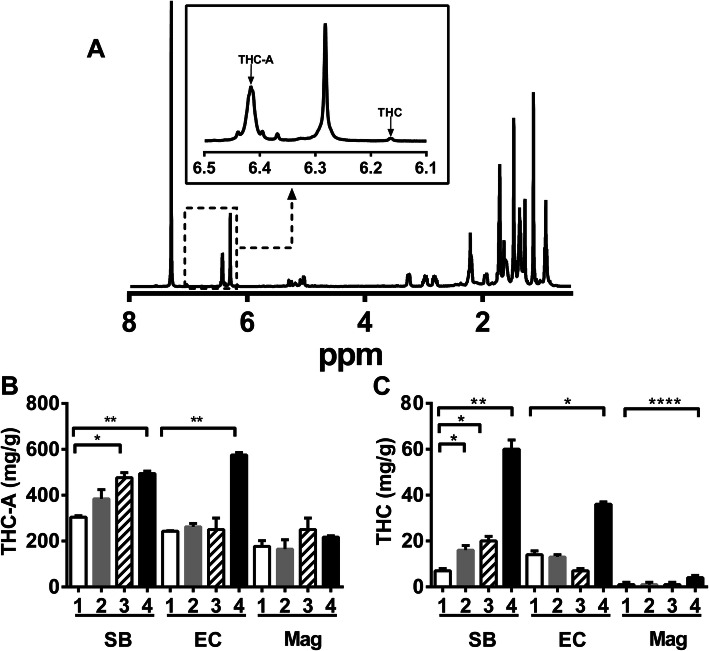
^1^H-RMN spectra and a zoom in showing the hydrogen signals used for quantification. Details of the spectra from SB3 is shown as an example. The H10: 6.39 ppm for THC-A and H2: 6.14 ppm for THC are shown (**a**). The amount of THC-A (**b**) and THC (**c**) per gram of extract determined by ^1^H-RMN from the inflorescences obtained during the first (white bars), second (grey bars), and third harvest (dashed bars) and after drying of the mature inflorescences (black bars). Statistically significant differences against the first harvest were found by one-way ANOVA followed by Sidak-Bonferroni multiple comparisons test (*p < 0.05, ** *p* < 0.01 **** *p* < 0.0001)

As expected, the selected extraction method (chloroform) lead to a cannabinoid rich profile with negligible evidence of other polyphenols. The quantification of phytocannabinoids was performed from specific signals in the ^1^H-NMR spectra, which in deuterated chloroform appeared between 5.5–6.8 ppm (Fig. 3). Peak areas were integrated with a chemical shift of 6.39 ppm for THC-A and 6.14 ppm for THC (Fig. 3), and 5.5–5.6 ppm for CBD and CBD-A, respectively (Hazekamp et al. 2004), using a known concentration of TMS as internal standard (Happyana and Kayser 2016). No further significant signals for other phytocannabinoids were registered. The predominant cannabinoid found in fresh extracts was the carboxylic acid of THC (THC-A, ≥95%) and in a very low proportion its decarboxylated product, in agreement with previous reports (Verhoeckx et al. 2006). This relationship between THC-A and THC levels was maintained during maturation in the fresh extracts of the analyzed varieties (Fig. 3). The two-way ANOVA showed a significant difference in composition among the varieties, a significant effect of time, and a significant interaction between time and variety. A progressive increase during maturation of THC-A was observed in the varieties SB, and Mag with *R*^*2*^ values of 0.89 and 0.46, respectively; while the increase of THC was only significant for SB (*R*^*2*^ = 0.79). The increase in the level of cannabinoids was congruent with previous reports for other THC-A/THC rich strains, where a significant increase in the accumulation of cannabinoids was found in the last 2 weeks before harvest (Happyana and Kayser 2016; Ingallina et al. 2020). The dried samples (collected at timepoint 4) from EC and SB showed the expected increase of the decarboxylated form (THC). However, the THC content of sample 4 in Mag remained constant (Fig. 3c). The content of cannabinoids in a fiber type (hemp) variety was also investigated. As expected, the analysis of the hemp extract showed the presence of CBD (198 mg/g) and CBD-A (92 mg/g) without the detectable presence of THC or THCA (≤ 1%).

### UV-Vis spectra

**Fig. 4:**
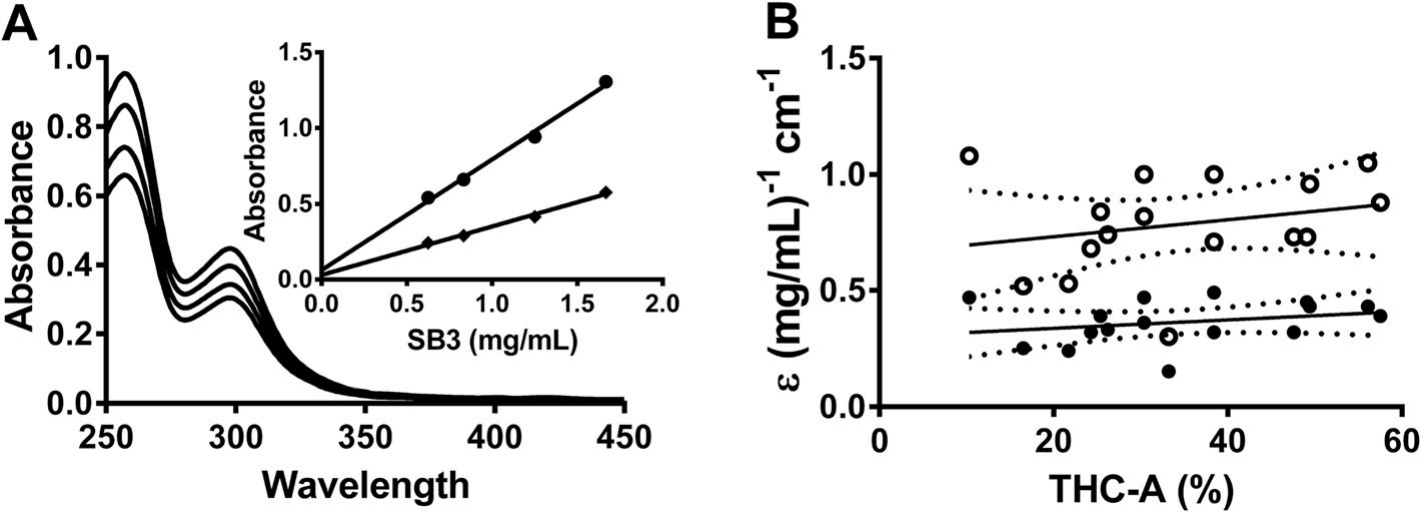
UV-Vis spectra of *Cannabis sativa* extracts. **a** The spectrum of the SB3 extract at 0.6; 0.8; 1.25; 1.7 mg/mL in 100 mM phosphate pH 7.4 is shown as an example. The inset shows the linear regression of absorbance at 257 nm (●) and 298 nm (♦) against the concentration of extract. **b** Correlation between the levels of THC-A determined by ^1^H-RMN and the extinction coefficients (**ε**) at 257 nm (○) and 298 nm (●) determined from the slope of plots as the one represented in the inset of (**a**)

Concordantly with the ^1^H-RMN results, the UV-Vis spectra of the extracts showed a scarce representation of most polyphenols, appearing only two areas of maximal absorbance at 298 and 257 nm (Fig. 4), coincident with the reported spectrum of pure THC-A (De Backer et al. 2009; Hazekamp and Fischedick 2012). The absorbance at those wavelengths increased linearly with the concentration of the extracts (Fig. 4, insert). The slopes of the calibration curves were in the range of 0.32–0.45 (mg/mL)^− 1^ cm^− 1^ at 298 nm and 0.52–1.1 (mg/mL)^− 1^ cm^− 1^ at 257 nm, being the ratio between both slopes ~ 2 for all samples (Table 2).

**Table 2:** Spectral characteristics of the extracts

Extract	257 nm		298 nm		Slope ratio (S_**257**_/S_**298**_)
	Slope (mg/mL)^**− 1**^	R^**2**^	Slope (mg/mL)^**− 1**^	R^**2**^	
**SB 1**	0.82 ± 0.06	0.999	0.36 ± 0.03	0.999	2.3
**SB 2**	0.71 ± 0.05	0.994	0.32 ± 0.03	0.994	2.2
**SB 3**	0.73 ± 0.04	0.99	0.32 ± 0.02	0.99	2.3
**SB 4**	0.96 ± 0.09	0.999	0.43 ± 0.03	0.999	2.2
**EC 1**	0.68 ± 0.05	0.998	0.32 ± 0.03	0.998	2.1
**EC 2**	0.74 ± 0.07	0.993	0.33 ± 0.03	0.993	2.2
**EC 3**	1.05 ± 0.09	0.999	0.43 ± 0.04	0.999	2.5
**EC 4**	0.98 ± 0.09	0.994	0.39 ± 0.04	0.994	2.3
**Mag 1**	0.84 ± 0.08	0.998	0.39 ± 0.04	0.998	2.2
**Mag 2**	0.52 ± 0.04	0.999	0.35 ± 0.02	0.999	2.1
**Mag 3**	0.73 ± 0.06	0.981	0.45 ± 0.03	0.981	1.6
**Mag 4**	0.53 ± 0.05	0.969	0.44 ± 0.02	0.969	2.2

From the UV/Vis spectra of the extracts, the presence of high concentrations of some phenolic compounds can be ruled out. For example, carotenoids including lutein, β-carotene and lycopene have maximal absorbance between 400 and 550 nm, with extinction coefficients in the range of 200–300 (mg/mL)^− 1^ cm^− 1^ (depending on the specific type and solvent) (Thrane et al. 2015), so even at low concentration they should be spectrophotometrically evident. Spectral evidences of flavonones such as quercetin and rutin (λmax = 257 mM^− 1^ and 370 nm, extinction coefficients 59 (mg/mL)^− 1^ cm^− 1^) or phenolic acids such as caffeic and chlorogenic acid (λmax = 325 nm) (Solovchenko 2010), were also absent in the analyzed extracts (Fig. 4). However, a very weak bivariate correlation (Pearson) between the extinction coefficients at 257 and 298 nm with the concentration of THC-A determined by ^1^H-NMR (*R*^*2*^ = 0.0058 and 0.07, respectively) were strongly indicative of the presence of other compounds interfering in the spectral analysis of the extracts (Fig. 4b). No correlations were observed also with the THC content (not shown).

### LDL oxidation

**Fig. 5:**
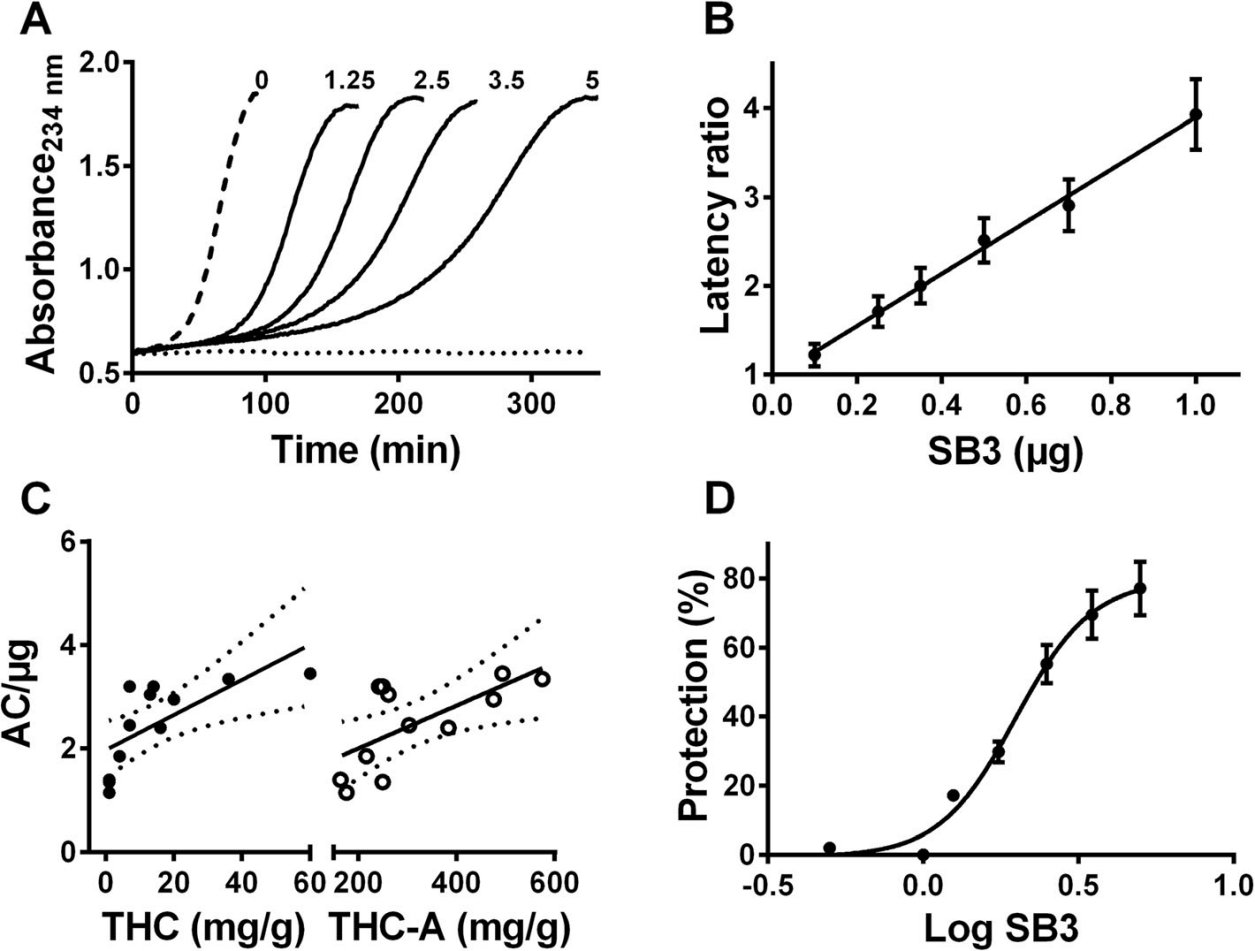
Conjugates dienes from LDL oxidation. **a** LDL (0.1 mg/mL) was exposed to CuSO_4_ (50 μM), and the formation of conjugated dienes was followed at 234 nm in the absence (dashed line) and the presence of cannabis extracts (continuous lines). Number over the curves represent the concentration of extract in μg/mL. Representative curves obtained in the presence of extracts from SB3 (1.25–5 μg/mL) are shown. The dotted line represents control LDL assayed in the same condition but without the addition of cupper ions. **b** Linear regression of latency ratios and the amount of SB3. The slope of these graphs (antioxidant capacity (AC/μg)) are summarized in Table 3. **c** Correlation between the AC/μg and the concentration of THC (*R*^*2*^ = 0.43). and THC-A (*R*^*2*^ = 0.49) in each extract. **d** Representative plot of percentage of protection, determined as described in Materials and Methods, against the concentration of SB3. Analogous plots obtained for each extract were used to obtain the values of IC_50_ shown in Table 3

**Fig. 6:**
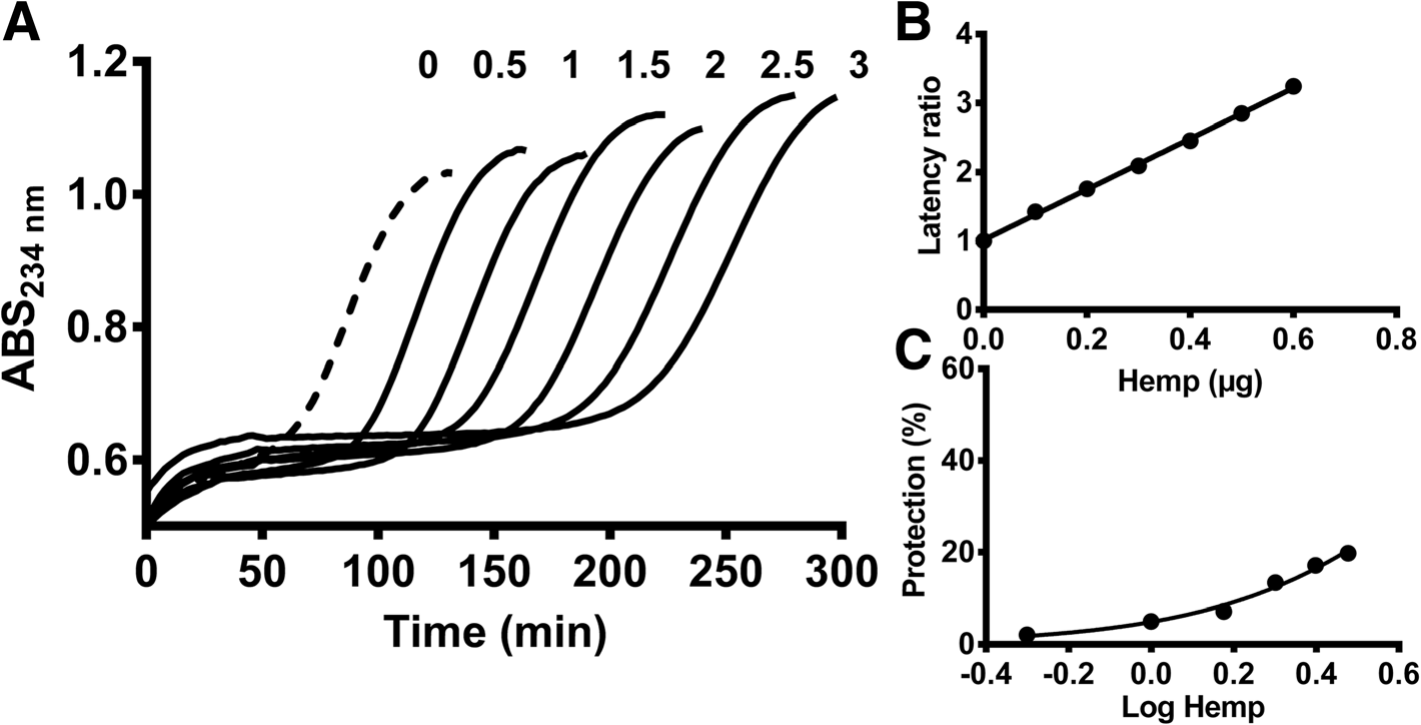
Effect of hemp extracts on LDL oxidation. **a** LDL was oxidized as in Fig. 5 and the time course of diene production was followed at 234 nm. Sigmoidal kinetics were obtained for the condition without (dashed line) and with hemp extracts (continuous lines). Number over the curves represent concentration of extract in μg/mL. **b** Linear regression of latency ratios against μg of hemp were used to calculate the AC/μg reported in Table 4. **c** A plot of percentage of protection against concentration was used to determine the IC_50_ reported in Table 4

The kinetic of generation of conjugated dienes from copper-treated LDL was assessed at 234 nm. As shown in Fig. 5a and S1–3 the initiation phase due to endogenous antioxidants lasted for half an hour or less in the absence of cannabinoids, while the duration of this initial phase increased linearly with the amount of extract present in the reaction vessel (Fig. 5b). In fact, the endogenous antioxidant capacity, responsible for the latency face, increased more than twice by each μg of SB (AC 2.4–3.5/μg) and EC (AC 3.2–3.4/μg) added. Meanwhile Mag, the sample with the lowest content of cannabinoids, showed an almost marginal effect (AC 1.2–1.9/μg) (Table 3). A significant correlation between the antioxidant capacity and the concentration of THC and THC-A was observed with R^2^ values of 0.43 and 0.49, respectively (Fig. 5c). The propagation phase was also affected by the presence of the extracts, with a significant decrease in the rate of formation of conjugated dienes (Fig. 5a). The decrease in the slope of the propagation phase give rise to IC_50_ values as low as 1.7–4.6 μg/mL (Table 3). The most mature samples achieved a higher antioxidant capacity per microgram of extract added, with lower IC_50_. However, this parameter showed a very poor correlation with the concentration of THC-A and THC (not shown). Meanwhile, an extract obtained from a hemp sample was also highly effective in prolonging the latency phase (AC 3.7 ± 0.1 /μg) (Fig. 6). The Hemp extract also decreased the rate of the propagation phase, but with a lower efficiency than the THC-A rich extracts (IC_50_ ~ 8 μg/mL) (Table 4). To further investigate the participation of the phytocannabinoids on the antioxidant properties of the extracts, the oxidation of LDL was analyzed in the presence of THC, THC-A, CBD and CBD-A. THC, CBD-A and CBD behave as highly effective antioxidants with AC/μg indexes of 21 ± 2, 9.4 ± 0.8 and 4.8 ± 0.1, respectively (Table 4). Conversely, THC-A showed a negligible influence on the latency phase of LDL oxidation with an AC/μg of 1.6 ± 0.5. The propagation phase of the lipid oxidation process remained unchanged in the presence of THC-A, the main cannabinoid present in SC, EC and Mag. Similar results were obtained with the main cannabinoids present in hemp, CBD and CBD-A. The slope of the fast increase in conjugated dienes production was decreased only by THC with an IC_50_ of 0.33 μg/mL (95% confidence interval 0.32–0.37 μg/mL).

**Table 3:** Effect on LDL oxidation

Sample	AC/μg^**a**^			IC_**50**_^**b**^ (μg/mL)		
	SB	EC	Mag	SB	EC	Mag
**1**	2.5 ± 0.2	3.2 ± 0.2	1.2 ± 0.2	2.6 (2.1–3.1)	4.6 (3.4–6.0)	2.1 (1.9–2.3)
**2**	2.4 ± 0.1	3.1 ± 0.1	1.4 ± 0.1	3.1 (2.8–3.4)	3.8 (2.7–5.3)	2.2 (1.8–2.6)
**3**	3.0 ± 0.1	3.2 ± 0.2	1.4 ± 0.3	2.5 (2.2–2.8)	2.6 (2.3–2.9)	2.2 ± (2.0–2.3)
**4**	3.5 ± 0.3	3.4 ± 0.1	1.9 ± 0.3	1.7 (1.4–2.2)	2.3 (1.9–2.8)	2.2 ± (2.0–2.4)

^a^The antioxidant capacity (AC) of the extracts was obtained from the slope of the plots of latency ratios against extract in μg and presented as mean ± SD (Fig. 5b). ^b^IC_50_ for the inhibitory effect on the propagation phase of LDL oxidation were obtained by non-linear fit of graphs as the one shown in Fig. 5d. As the fits are log transformed the estimates for the IC_50_ are presented with the 95% confidence intervals

**Table 4:** Effect of hemp and phytocannabinoids on LDL oxidation

Sample	AC/μg^a^	IC_**50**_ (μg/mL)^**b**^
**Hemp**	3.7 ± 0.1	8.0 (5.6–10.2)^d^
**THC-A**	1.6 ± 0.5	NP ^c^
**THC**	21 ± 2	0.33 (0.32–0.37)^d^
**CBD-A**	9.4 ± 0.8	NP
**CBD**	4.8 ± 0.1	NP

^a^Antioxidant capacity per microgram (AC/μg) was obtained from the slope of latency ratio vs. μg of sample. ^b^IC_50_ values were obtained from plots of protection against the log of concentration (Fig. 6 and S4). ^c^NP, no protection. ^d^As the fits to calculate IC_50_ are log transformed the estimates for the IC_50_ are presented as mean with the 95% confidence intervals

## Discussion

The oxidation of LDL in the dysfunctional subendothelial space is one of the events recognized as instrumental in foam cell formation and the evolution of the atherosclerotic lesion. Kinetic and molecular components involved in LDL oxidation have been extensively described (Jürgens et al. 1987). This process can be triggered by different stimulus, including enzymes and metal ions, leading to both lipid and protein (Apo-B100) oxidation. Lipid oxidation can be easily followed by measuring the time course of the formation of conjugated dienes. The cascade of events provided the basis for a model that allows for testing the ex vivo resistance of human LDL isolated from plasma, when exposed to Cu^2+^ ions as a pro-oxidant under standardized conditions (Esterbauer et al. 1989; Kiokias et al. 2018). This kinetic profile shows a latency, a propagation and a termination phase (Fig. 1). The latency phase is attributed to the presence of endogenous antioxidants in the LDL particles. These antioxidants are accountable for scavenging the free radicals triggers of the initiation reactions of the lipoperoxidation process (Cadenas and Sies 1998). On the other hand, the overall time course of lipid peroxidation, including propagation, is largely influenced by the rate constants for propagation reactions and termination involving radical recombination (Cadenas and Sies 1998). Hence, the differential effect on both parameters could be explaining differential mechanisms of antioxidant action.

Given the relevance of LDL oxidation in the development of atheromatous plaques, and the urgent need of appropriate therapies to ameliorate and prevent its progression, the potential of *Cannabis sativa* extracts to interfere with the oxidation of LDL was explored. Independently of the predominant cannabinoid composition, THC-A/THC-rich or CBD-A/CBD rich extracts, the whole extracts used have the ability to inhibit both phases of LDL oxidation, suggesting a relevant potential of medicinal cannabis as a supplement in atherosclerosis therapy. In addition, most of the isolated phytocannabinoids analyzed acted as antioxidants, prolonging the latency phase of lipid oxidation, but were unable to interfere with the propagation phase. THC was the only isolated compound able to prolog the latency phase and to break the lipid oxidation chains induced by Cu^2+^ in LDL lipids. Although THC increased during the maturation period (samples 1 through 3) and in the dried samples (samples 4), a moderate or absent correlation was seen between the concentration of this compound in the extracts and the antioxidant capacity or protection. These results suggest that the maturation of the inflorescences lead to an increase in the components able to decrease the oxidation of the lipids present in LDL. This tendency, along with the fact that the concentration of cannabinoids present in the extracts increases as the maturation of the samples progresses, suggests that cannabinoids contribute, at least in part, to the protective effects observed. However, since the correlation between the antioxidant parameters and the concentration of THC-A and THC was low, and the lack of protection by THC-A in both phases and of CBD-A and CBD during the propagation phase point towards the contribution of other components present in the extracts.

Chemically the antioxidant capacity of phytocannabinoids depends on the ability of the phenol group to transfer hydrogen atoms or electrons to the oxidant. Other functional groups surrounding the phenol are responsible for differences in the nucleophilicity between phenolic compounds through inductive or resonance effects. In that sense, THC was the more effective antioxidant prolonging the latency phase of lipid oxidation, in agreement with previous in silico analysis, predicting a higher antioxidant potency for THC than for CBD (Borges et al. 2013). The same work described the formation of a stable semiquinone radical after scavenging the free-radical species by THC, with an important role at the propagation and termination steps of oxidation (Borges et al. 2013). In fact, THC behaves exactly as predicted by breaking the lipoperoxidation chains in LDL (Fig. S4 and Table 4), supporting its role as antioxidant and also as an effective chain breaking reagent. The inhibitory effect of THC on LDL oxidation can curb the spread of foam cells into the lesion and impede oxLDL to act as a danger associated molecular pattern, decreasing the inflammatory response related to the development of the atheromatous plaque (Geovanini and Libby 2018). In atherosclerosis medicinal cannabis appears as a multi target therapy, due also to the already known anti-inflammatory properties of cannabinoids. In this regard, cannabinoids present a relevant inhibitory effect on pro-inflammatory mediators as TNF-α and COX-2 (Takeda et al. 2008; Verhoeckx et al. 2006). In addition, endogenous and exogenous cannabinoids bind and activate PPARγ (O’Sullivan 2015, 2016), being this mechanism responsible for the neuroprotective effect observed (Nadal et al. 2017). In the cardiovascular system a vasorelaxant effect of cannabinoids mediated by PPARγ and inhibited by catalase and superoxide dismutase was described (O’Sullivan et al. 2006), pointing to the participation of cannabinoids in the regulation of cell signaling through both macromolecules and reactive oxygen species, supporting the existence of multiple sites and mechanisms of action for the different cannabinoids. Finally, is important to understand that there is an urgent need of scientific knowledge to enlighten the decision making about the use of phytocannabinoids or other substances able to modulate the endocannabinoid system, including the exact composition, biological effects and action mechanisms.

This study is not without limitations. Taking into account the hypothesis of “entourage effect” (González-Burgos and Gómez-Serranillos 2012; Radwan et al. 2009), in which cannabis is characterized as a synergistic set of compounds, it is possible that molecules other than phytocannabinoids present in whole cannabis extracts, such as terpenes and other organic compounds, may be acting in synergy with the most abundant cannabinoid, in this case THC-A. However, in this study it was not possible to identify the presence of further compounds, cannabinoids or terpenes, an issue that will be addressed in future studies. On the other hand, the in vivo mechanisms of LDL oxidation are more complex than the well-characterized ex vivo Cu^2+^-induced oxidation. Nonetheless, the effect of many potential therapeutics on the ex vivo Cu^2+^-induced oxidation of LDL has informed human studies. Moreover, not all compounds that protect ex vivo, have shown promise in human studies (reviewed in (Winklhofer-Roob et al. 2017)). Hence, it is possible that phytocannabinoids protect LDL from oxidation, but they might not offer protection from atherosclerosis development. This fact highlights the need to increase the complexity of the experimental models to test the potential therapeutic effect of cannabinoids. In light of this, it is important to acknowledge that the effects of cannabinoids on the cells involved in the development of atherosclerosis were not assessed in this study, and are the subject of ongoing studies in the lab.

## Conclusions

Our findings support the beneficial effects of *Cannabis sativa* extracts on the initial phase of atherosclerosis. Since isolated cannabinoids were less effective preventing the oxidation of LDL, a synergistic effect between the diverse arrange of phytochemicals present in complex extracts is supported, reinforcing the entourage hypothesis and the use of whole medicinal cannabis extracts for therapeutic purposes.

## Supplementary information

**Additional file 1: Figure S1.** Effect of SB on LDL oxidation. LDL oxidation by copper (II) ions, in the absence (black line) and in the presence of increasing concentrations of the extracts (referenced with color codes to μg/mL on the right of each graph) obtained from the different maturation stages (samples 1, 2 and 3) and from a dried sample (4) of the SB variety was followed at 234 nm. **Figure S2.** Effect of EC on LDL oxidation. LDL oxidation by copper (II) ions, in the absence (black line) and in the presence of increasing concentrations of the extracts (referenced with color codes to μg/mL on the right of each graph) obtained from the different maturation stages (samples 1, 2 and 3) and from a dried sample (sample 4) of the EC variety was followed at 234 nm. **Figure S3.** Effect of Mag on LDL oxidation. LDL oxidation by copper (II) ions, in the absence (black line) and in the presence of increasing concentrations of the extracts (referenced with color codes to μg/mL on the right of each graph) obtained from the different maturation stages (samples 1, 2 and 3) and from a dried sample (sample 4) of the Mag variety was followed at 234 nm. **Figure S4.** Effect of phytocannabinoids on conjugated dienes formation. **A**. LDL (0.1 mg/mL) was exposed to CuSO4 (50 μM), and the formation of conjugated dienes was followed at 234 nm in the absence (dashed line) and the presence of increasing concentrations of phytocannabinoids as stated on the figures. **B**. Latency ratios were represented as a function of the cannabinoid added. The slope of these graphs (Antioxidant capacity) for each compound are summarized in Table 4. **D**. Percentage of protection, determined as described in Materials and Methods, against the log of cannabinoid concentration.

## Data Availability

Within the experiments presented in this paper, we have available all the necessary data and they are available from the corresponding author upon reasonable request.

## Abbreviations

ANOVA: Analysis of variance
CBD: Cannabidiol, IUPAC name: 2-[(1R,6R)-3-methyl-6-prop-1-en-2-ylcyclohex-2-en-1-yl]-5-pentylbenzene-1,3-diol
CBD-A: Cannabidiolic acid, IUPAC name: 2,4-dihydroxy-3-[(1R,6R)-3-methyl-6-prop-1-en-2-ylcyclohex-2-en-1-yl]-6-pentylbenzoic acid
CBR2: Cannabinoid receptor 2
CDCl_3_: Deuterated chloroform
CHCl_3_: Chloroform
COX-2: Cyclooxygenase-2
CuSO_4_: Copper sulfate
DMSO: Dimethyl sulfoxide
IC_50_: Inhibitory Concentration 50%
IL-10: Interleukin 10
IL-12: Interleukin 12
KBr: Potassium bromide
LDL: Low-density lipoprotein
NaCl: Sodium chloride
NMR: Nuclear Magnetic Resonance
oxLDL: Oxidized low-density lipoprotein
PPAR-γ: Peroxisome proliferator-activated receptor gamma
PUFA: Polyunsaturated fatty acid
PW: Pulse width
RD: Relaxation time
THC: Δ^9^-tetrahydrocannabinol, IUPAC Name: (6aR,10aR)-6,6,9-trimethyl-3-pentyl-6a,7,8,10a-tetrahydrobenzo [c]chromen-1-ol
THC-A: Δ^9^-tetrahydrocannabinolic acid, (6aR,10aR)-1-hydroxy-6,6,9-trimethyl-3-pentyl-6a,7,8,10a-tetrahydrobenzo [c]chromene-2-carboxylic acid
TMS: Tetramethylsilane
TNF-α: Tumor necrosis factor alpha
