# Supplementary material for: Cannabis sativa extracts protect LDL from Cu2+-mediated oxidation
Source: J Cannabis Res. 2020 Oct 15;2:37. doi: 10.1186/s42238-020-00042-0 (PMC7592720; doi:10.1186/s42238-020-00042-0)
Supplement: Supplementary file 1 — Additional file 1: Figure S1. Effect of SB on LDL oxidation. LDL oxidation by copper (II) ions, in the absence (black line) and in the presence of increasing concentrations of the extracts (referenced with color codes to μg/mL on the right of each graph) obtained from the different maturation stages (samples 1, 2 and 3) and from a dried sample (4) of the SB variety was followed at 234 nm. Figure S2. Effect of EC on LDL oxidation. LDL oxidation by copper (II) ions, in the absence (black line) and in the presence of increasing concentrations of the extracts (referenced with color codes to μg/mL on the right of each graph) obtained from the different maturation stages (samples 1, 2 and 3) and from a dried sample (sample 4) of the EC variety was followed at 234 nm. Figure S3. Effect of Mag on LDL oxidation. LDL oxidation by copper (II) ions, in the absence (black line) and in the presence of increasing concentrations of the extracts (referenced with color codes to μg/mL on the right of each graph) obtained from the different maturation stages (samples 1, 2 and 3) and from a dried sample (sample 4) of the Mag variety was followed at 234 nm. Figure S4. Effect of phytocannabinoids on conjugated dienes formation. A. LDL (0.1 mg/mL) was exposed to CuSO4 (50 μM), and the formation of conjugated dienes was followed at 234 nm in the absence (dashed line) and the presence of increasing concentrations of phytocannabinoids as stated on the figures. B. Latency ratios were represented as a function of the cannabinoid added. The slope of these graphs (Antioxidant capacity) for each compound are summarized in Table 4. D. Percentage of protection, determined as described in Materials and Methods, against the log of cannabinoid concentration. [file 42238_2020_42_MOESM1_ESM.docx]

**Figure S1. Effect of SB on LDL oxidation.** LDL oxidation by copper (II) ions, in the absence (black line) and in the presence of increasing concentrations of the extracts (referenced with color codes to µg/mL on the right of each graph) obtained from the different maturation stages (samples 1, 2 and 3) and from a dried sample (4) of the SB variety was followed at 234 nm.

**Figure S2. Effect of EC on LDL oxidation.** LDL oxidation by copper (II) ions, in the absence (black line) and in the presence of increasing concentrations of the extracts (referenced with color codes to µg/mL on the right of each graph) obtained from the different maturation stages (samples 1, 2 and 3) and from a dried sample (sample 4) of the EC variety was followed at 234 nm.

**Figure S3. Effect of Mag on LDL oxidation.** LDL oxidation by copper (II) ions, in the absence (black line) and in the presence of increasing concentrations of the extracts (referenced with color codes to µg/mL on the right of each graph) obtained from the different maturation stages (samples 1, 2 and 3) and from a dried sample (sample 4) of the Mag variety was followed at 234 nm.

**Figure S4. Effect of phytocannabinoids on conjugated dienes formation. A.** LDL (0.1 mg/mL) was exposed to CuSO_4_ (50 μM), and the formation of conjugated dienes was followed at 234 nm in the absence (dashed line) and the presence of increasing concentrations of phytocannabinoids as stated on the figures. **B.** Latency ratios were represented as a function of the cannabinoid added. The slope of these graphs (Antioxidant capacity) for each compound are summarized in Table 4. **D.** Percentage of protection, determined as described in Materials and Methods, against the log of cannabinoid concentration**.**
